# Piezo1 channels mediate vasorelaxation of uterine arteries from pseudopregnant rats

**DOI:** 10.3389/fphys.2023.1140989

**Published:** 2023-06-01

**Authors:** Olufunke O. Arishe, Jaine McKenzie, Vanessa Dela Justina, Raiana Dos Anjos Moraes, R. Clinton Webb, Fernanda Priviero

**Affiliations:** ^1^ Department of Physiology, University of Tennessee Health Sciences Center, Memphis, TN, United States; ^2^ Department of General Surgery, Vanderbilt University, Nashville, TN, United States; ^3^ Graduate Program in Biological Sciences, Federal University of Goias, Goiânia, Goias, Brazil; ^4^ Department of Cell Biology and Anatomy—School of Medicine, University of South Carolina, Columbia, SC, United States; ^5^ Cardiovascular Translational Research Center—School of Medicine, University of South Carolina, Columbia, SC, United States; ^6^ College of Engineering and Computing, Biomedical Engineering Program, University of South Carolina, Columbia, MO, United States

**Keywords:** Piezo1 channel, vasorelaxation, pregnancy, endothelium, mechanotransduction

## Abstract

**Introduction:** There is a great increase in uterine arterial blood flow during normal pregnancy, which is a result of the cardiovascular changes that occur in pregnancy to adapt the maternal vascular system to meet the increased metabolic needs of both the mother and the fetus. The cardiovascular changes include an increase in cardiac output and more importantly, dilation of the maternal uterine arteries. However, the exact mechanism for the vasodilation is not fully known. Piezo1 mechanosensitive channels are highly expressed in endothelial and vascular smooth muscle cells of small-diameter arteries and play a role in structural remodeling. In this study, we hypothesize that the mechanosensitive Piezo1 channel plays a role in the dilation of the uterine artery (UA) during pregnancy.

**Methods:** For this, 14-week-old pseudopregnant and virgin Sprague Dawley rats were used. In isolated segments of UA and mesenteric resistance arteries (MRA) mounted in a wire myograph, we investigated the effects of chemical activation of Piezo1, using Yoda 1. The mechanism of Yoda 1 induced relaxation was assessed by incubating the vessels with either vehicle or some inhibitors or in the presence of a potassium-free physiological salt solution (K^+^-free PSS).

**Results:** Our results show that concentration-dependent relaxation responses to Yoda 1 are greater in the UA of the pseudo-pregnant rats than in those from the virgin rats while no differences between groups were observed in the MRAs. In both vascular beds, either in virgin or in pseudopregnant, relaxation to Yoda 1 was at least in part nitric oxide dependent.

**Discussion:** Piezo1 channel mediates nitric oxide dependent relaxation, and this channel seems to contribute to the greater dilation that occurs in the uterine arteries of pseudo-pregnant rats.

## 1 Introduction

In normal pregnancy, there is an increase in maternal uterine artery blood flow to the feto-placenta unit, and this results from increases in maternal blood volume coupled with a decrease in vascular resistance (Ko et al., 2018). Cardiovascular changes occur in pregnancy to adapt the maternal system to the increased blood flow to meet the increased metabolic needs of both the mother and the fetus. This increase in blood flow is necessary for the development of the fetus and ultimately may contribute to a greater birth weight (Julian et al., 2009). Increases in blood flow stimulate dilation and remodeling of the uterine arteries leading to an increase in the diameter of the uterine artery to accommodate this large increase in blood flow to the feto-placenta unit (Koga et al., 2003). The underlying mechanisms that modulate uterine vascular function in pregnancy are currently incompletely understood.

Blood flow is controlled in all vascular beds by autoregulatory mechanisms (Iring et al., 2019). During pregnancy, blood flow greatly increases in the uterine vasculature to about double the flow in the non-pregnant uterine vasculature. Despite this increase in blood flow, blood pressure is not increased, instead, there is increased dilation of the uterine arteries to maintain this increase in blood flow. This hemodynamic adaptation during normal pregnancy is important for the maintenance of the mother and fetus, and any disorder or abhorrent hemodynamic adaption will lead to placental ischemia which results in widespread dysfunction of maternal endothelium, activation of vasoconstriction, and development of hypertension (Sanghavi and Rutherford, 2014).

Piezo channels are mechanosensitive cation-selective channels. They are large proteins with over 2000 amino acids. They are trimers shaped like a propeller with three blades organized around a central pore (Coste et al., 2010). This helps to preserve the properties of the channel under various membrane tensions (Ranade et al., 2014). Activation of Piezo channels generates a cationic non-selective current and the channels are permeable to Na^+^, K^+^, Ca^2+^ and Mg^2+^ (Parpaite and Coste, 2017).

Piezo1 channels are highly expressed in vascular smooth muscle cells of small-diameter arteries and play a role in the structural remodeling of the arteries. Studies have also shown that Piezo1 is present in uterine arteries, and they are not exclusive to the endothelial cells (John et al., 2018).

Piezo proteins have large molecular weights and do not conform structurally to other known mammalian proteins or channels (Gnanasambandam et al., 2015; Lewis and Grandl, 2015). This feature has made it difficult to study the mechanism by which the channel senses mechanical stimuli, how they interact with downstream signaling pathways. Another feature of Piezo channels is that when the channels are activated by mechanical stimuli, they mediate immediate increases in currents which decline even in the presence of the stimulus. This leads to rapid inactivation in a voltage-dependent manner and the molecular mechanism for this inactivation is currently unknown (Lewis and Grandl, 2015; Wu et al., 2017). Some studies have been carried out in an attempt to elucidate these mechanisms of Piezo1 channel action. Gnanasambandam et al. reported that the Piezo 1 channel is permeable to the monovalent cations: K^+^, Na^+^, Cs^+^, and Li^+^ and to the di-valent cations: Ba^2+^, Ca^2+^, and Mg^2+^ but it is not permeable to Mn^2+^ (Gnanasambandam et al., 2015). As the concentration of these ions increases intracellularly, they stimulate/activate various intracellular signaling pathways. Another study reported that activation of Piezo1 channel increases intra-cellular Ca^2+^ levels stimulating the activity of transglutaminases that cross-link enzymes required for remodeling of small arteries (Retailleau et al., 2015). Moreover, a previous study demonstrated that Yoda1 increased the intracellular concentration of calcium in the endothelial cell, and induced vasodilation of uterine arteries with higher magnitude of relaxation in arteries from late pregnant rats, compared to non-pregnant rats. Flow-induced vasodilation was decreased in the presence of L-NAME, and virtually abolished in the presence of GsMTx-4 (14), suggesting that Piezo1 activation induces relaxation not only through NO release. Because potassium channels play an important role in the relaxation of uterine arteries, and its deficiency is also associated with complications in pregnancy (Bresnitz and Lorca, 2022), we sought to further investigate whether Yoda1-induced relaxation was associated with potassium channels activation.

**FIGURE 1:**
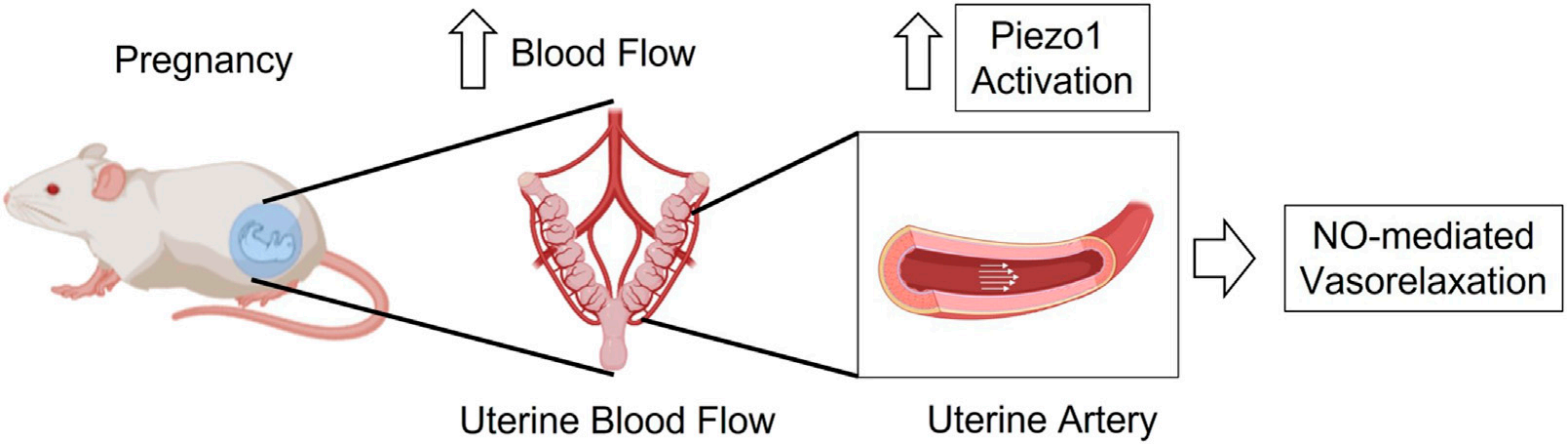
*Graphical hypothesis* **–**In pregnancy, activation of Piezo1 channel is increased in the UA to induce vasorelaxation through a mechanism that is at least in part dependent of nitric oxide.

Therefore, in this study, we hypothesize that the shear-stress-associated mechanosensitive Piezo1 channel is upregulated in the uterine arteries during pregnancy and therefore, Piezo1 activation might be impaired in hypertensive pregnancy (Figure 1). In addition, we aimed to investigate the underlying mechanisms of action of Yoda1, upon activation of Piezo1 in the uterine arteries of pseudo-pregnant rats.

## 2 Materials and methods

### 2.1 Animals

Twelve-week-old virgin female Sprague-Dawley and age-matched male Sprague-Dawley rats were used for the study. They were purchased from Envigo RMS, Inc. And all rats were maintained in GM500 individually ventilated cages (Animal Care Systems), at 21°C, 50%–70% humidity, on a 12-h light/dark cycle, chow and water were made available *ad libitum*. All animal procedures were performed at the University of South Carolina and were conducted in accordance with the Guide for the Care and Use of Laboratory Animals of the National Institutes of Health (NIH) and ethical standards of Institutional Animal Care and Use Committee of the University of South Carolina.

### 2.2 Pseudopregnancy

Some female rats were allowed to acclimatize for 1 week after which a normal estrous cycle was determined using vaginal cytology, and then the rats in the pro-estrous/estrous phase of their cycle were chosen to mate with their age-matched vasectomized males. Following overnight mating, the females were taken out and placed in separate cages and that day was noted to be day 1 of pseudopregnancy. Rats were anesthetized and euthanized on days 9–11 of pseudopregnancy. Pseudopregnant rats were used in our study because pseudopregnant rodents have been used as a pregnancy-like model, which can be manipulated without terminating the pregnancy or killing the fetuses, pseudopregnant rats have the same hormonal profile and vascular hemodynamic adaptations as pregnant rats (Bui et al., 1986; van der Heijden et al., 2005; Augustine and Grattan, 2008; Augustine et al., 2019).

### 2.3 Pregnancy

Females in the pro-estrous/estrous phase of their cycle were chosen to mate with age-matched males. Following overnight mating, vaginal smears was inspected for the presence of spermatozoa using light microscopy, the morning which spermatozoa is found was day 1 of pregnancy.

### 2.4 Hypertension in pregnancy

Hypertension was induced by treating the pregnant rats with synthetic CpG ODN (ODN 2395; Invitrogen, San Diego, CA) via three intraperitoneal injections (100 µg/rat) while the normotensive controls were treated with saline (vehicle) on the 14th, 17th and 18th days of pregnancy. A similar, time-controlled, treatment regimen was used for non-pregnant rats. Mean Arterial pressure (MAP) was measured, tissue harvested, and vascular experiments were conducted on gestational day 20.

### 2.5 Blood pressure measurements

MAP was measured in the rats under anaesthesia (isoflurane 1.5%, via inhalation). The animal was carefully dissected to expose the femoral artery; the catheter was inserted into the femoral artery while the other end was connected to the pressure transducer coupled to a PowerLab system (PowerLab 4SP/ML750).

### 2.6 Vascular function experiments

Virgin, pseudopregnant, and pregnant rats were anesthetized with isoflurane *via* a nose cone for surgical procedures (5% in 100% oxygen) and euthanized by thoracotomy and exsanguination *via* cardiac puncture. The uterine horns and small intestine with the attached vasculature were excised and placed in 4°C physiological salt solution (PSS) containing (mmol/L): NaCl (130), NaHCO_3_ (14.9), KCl (4.7), KH_2_PO4 (1.18), MgSO_4_.7H_2_O (1.18), CaCl_2_.2H_2_O (1.56), ethylenediaminetetraacetic acid [EDTA (0.026)], and glucose (5.5) (all Sigma-Aldrich). Uterine and mesenteric resistance arteries were carefully cleaned of adhering perivascular adipose tissues under light microscopy in ice-cold PSS. The uterine artery of one uterine horn and second-order mesenteric resistance arteries were cut into 2 mm segments.

The arterial rings were mounted on DMT wire myographs using 40-µm wires (Danish Myo Tech, Aarhus, Denmark) and were normalized to their optimal lumen diameter for active tension development, which was determined based on the internal circumference (*L*_*0*_) t0 90% of what the vessels would have if they were exposed to a passive tension equivalent of 100 mmHg (*L*_*100*_) transmural pressure (Mulvany and Aalkjaer, 1990). The diameter (*I*_*1*_) was then determined according to the equation *(I1)* = *L*_*1*_/π, using the software specifically for the normalization of resistance arteries (DMT Normalization Module; LabChart v.5.5.6, AD Instruments). Arteries were then bathed in PSS maintained at 37°C, bubbled continuously with 5% CO_2_ and 95% O_2_ for 30 min, and rinsed three times at 10-min intervals. Thereafter, the vessels were initially contracted with 120 mM KCl PSS (the vessels were considered viable only if they contracted to a force greater than 10 mN in response to 120 mM KCl). Endothelium integrity was then tested by contracting the arterial rings with 3 × 10^−6^ M phenylephrine (PE) followed by relaxation with 3 × 10^−6^ M acetylcholine (ACh) and the endothelium was considered intact if arterial rings relaxed more than 70% to ACh. Arterial rings that relaxed less than 70% to ACh were not considered for the study. Arterial rings were washed with fresh PSS three times and rested for 10 min after which they were pre-contracted with 3 × 10^−6^ M PE, and concentration-response curves to Yoda1 (a chemical activator of Piezo1 channel) were obtained in both MRAs and UAs of virgin, pseudopregnant, and pregnant rats. The mechanism of Yoda1-induced relaxation was assessed by incubating the vessels with either vehicle or one of the following inhibitors: L-NAME (10^−4^ M; nitric oxide synthase inhibitor), indomethacin 10^−5^ M (cyclooxygenase (COX) 1 and 2 inhibitor), tetraethylammonium (TEA 2 × 10^−3^ M; non-selective potassium channel inhibitor); charybdotoxin (ChTX 10^−7^ M; a blocker of the intermediate conductance Ca^2+^ activated K^+^ channel (IKCa)), and apamin (Ap 10^−9^ M, a blocker of the small conductance Ca^2+^ activated K^+^ channel (SKCa) and in the presence of the potassium-free physiological salt solution (K^+^-free PSS).

### 2.7 Statistical analysis

Data are expressed as mean ± S.E.M. of three to five rats per group, as indicated in each dataset. Sample size was calculated using G*Power 3.1.9.7 (desired power of 0.95 with a probability of a Type I error of 0.05), which provided a *n* = 3 per experimental group as the minimum number of animals necessary to generate a statistically significant experimental outcome (*p* < 0.05). Relaxation responses were expressed as the percentage of decrease in the contraction induced by PE. The Shapiro-Wilk test was used to determine normal distribution. Statistical differences were calculated using Student’s t-test or two-way ANOVA with repeated measures. Significance was set at *p* < 0.05. All statistical tests were performed using GraphPad Prism (v. 9.5.1 GraphPad software).

## 3 Results

### 3.1 Vascular reactivity

#### 3.1.1 Concentration-dependent relaxation responses to Yoda1 are greater in uterine artery but not in MRA from pseudo-pregnant rats compared to those from virgin rats

**FIGURE 2:**
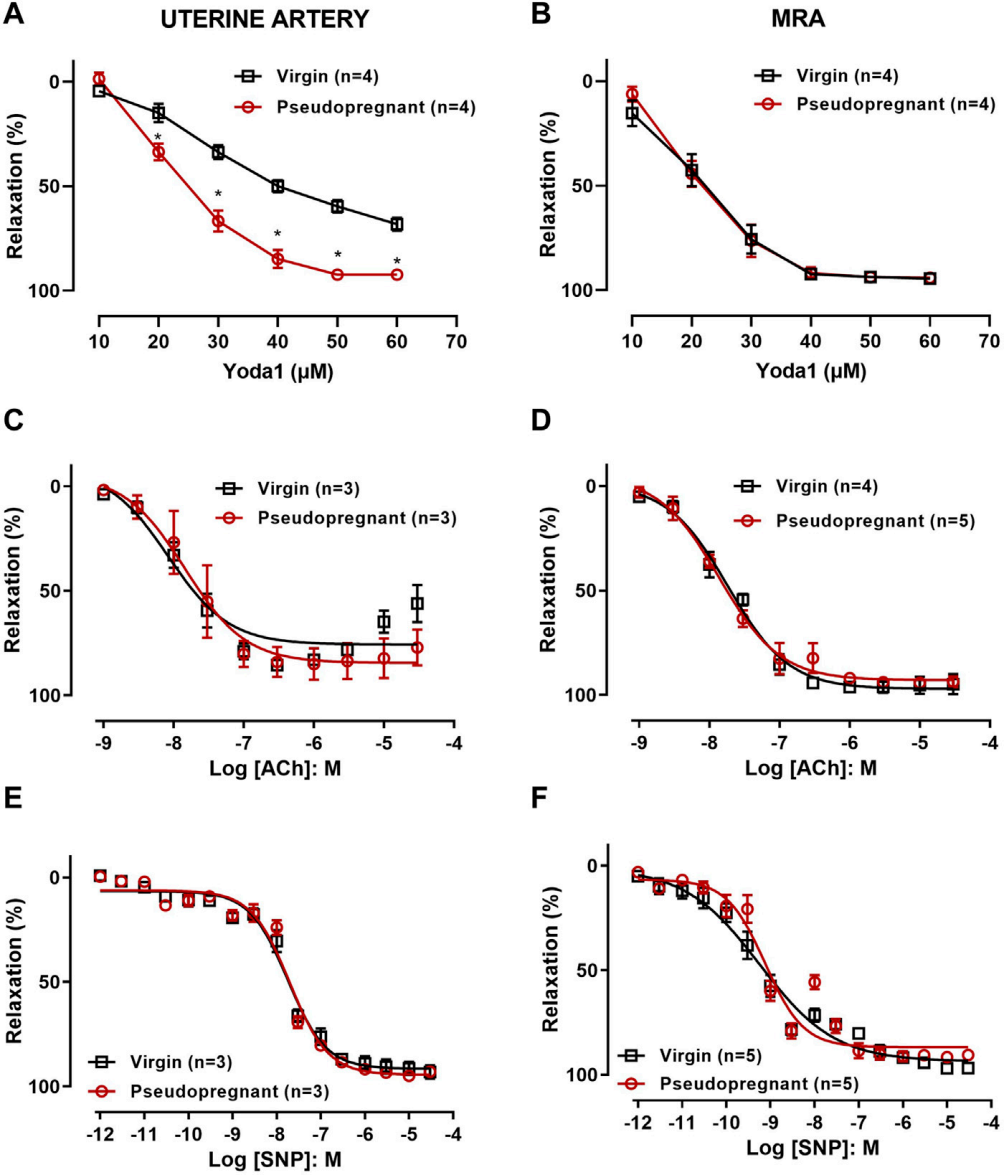
Concentration-dependent relaxation responses of uterine arterial ring or MRA to **(A,B)** Yoda1 [10^−5^ M - 6 × 10^−5^ M]; **(C,D)** ACh [10^−9^ M—3 × 10^-5^ M]; and **(E,F)** SNP [10^−12^ M—3 × 10^−5^ M]; Vessels were pre-contracted with 3 × 10^−6^ M phenylephrine. Mean ± S.E.M. of three to five rats. Responses to Yoda1 were analyzed using a two-way ANOVA test, while the responses to ACh and SNP were analyzed using Student’s t-test. *p < 0.05 compared to virgin rats.

The role of Piezo1 channels in the relaxation of the uterine artery during pregnancy was evaluated by obtaining concentration-response curves to Yoda1 [10^−5^ M—6 × 10^−5^ M] in the uterine arterial ring segments from non-pregnant and pseudopregnant rats. Our results (Figure 2A) show that Yoda1 relaxed the uterine arteries (UAs) from both virgin and pseudopregnant rats, however, with greater magnitude in UAs of the pseudopregnant rats compared to those from the virgin rats. In the mesenteric resistance artery (MRA), Yoda1 induced the relaxation to nearly 100% and, contrary to the observed in UA, the responses were not different between groups (Figure 2B). In addition, we performed concentration-response curves to ACh [10^−9^ M—3 × 10^−5^ M] (Figures 2C,D) and SNP [10^−12^ M—3 × 10^-5^ M] (Figures 2E,F). In both UA and MRA no differences in the relaxation responses to ACh and SNP were observed when comparing control with pseudopregnant rats.

#### 3.1.2 Concentration-dependent relaxation response to Yoda1 is nitric oxide-dependent

**FIGURE 3:**
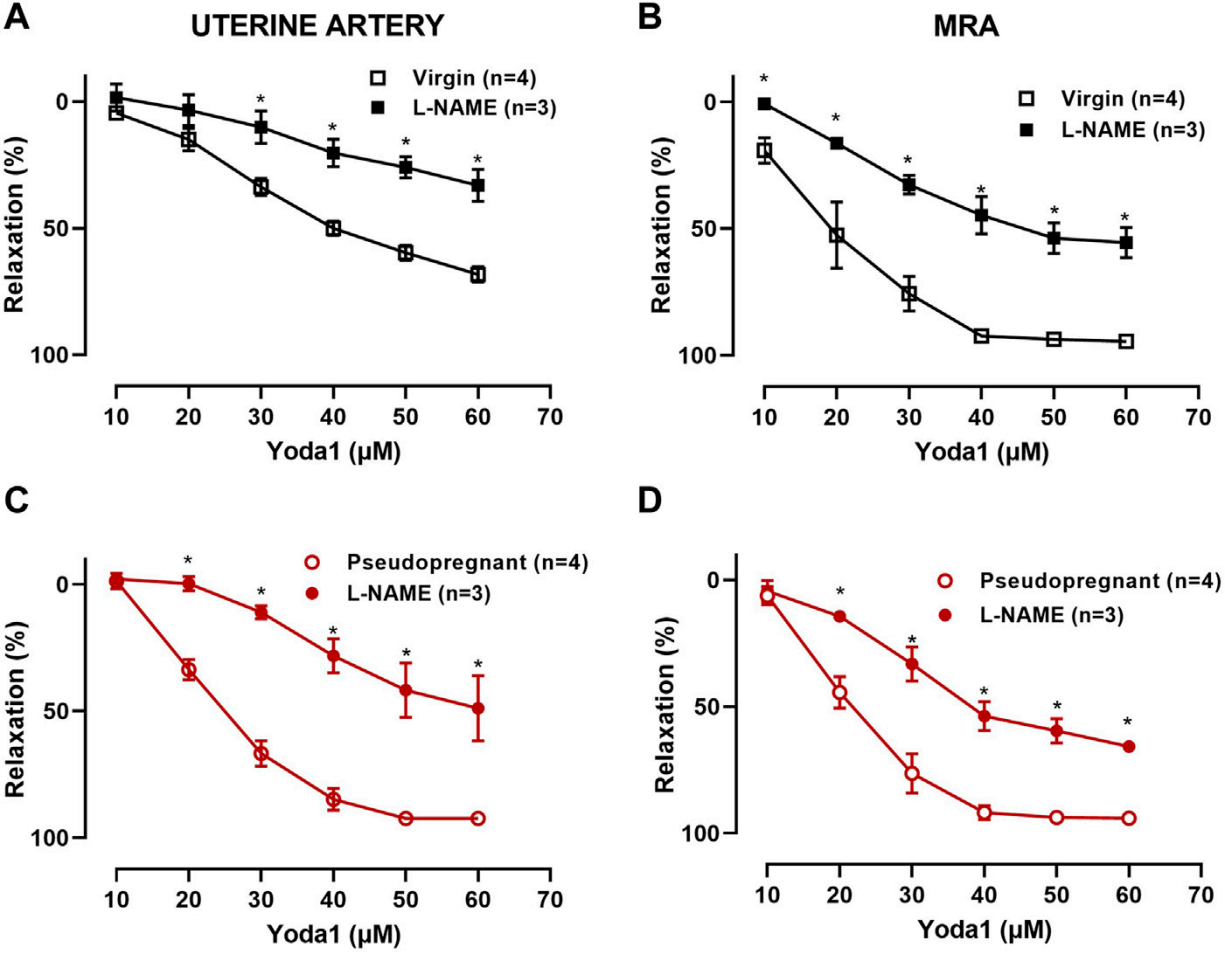
Concentration-dependent relaxation responses to Yoda1 [10^−5^ M—6 × 10^−5^ M] in UA and MRA in the presence or absence of 10^−4^ M L-NAME. Vessels were pre-contracted with 3 × 10^−6^ M PE. **(A)** Virgin uterine arteries; **(C)** Pseudopregnant uterine arteries; **(B)** Virgin mesenteric resistance arteries; **(D)** Pseudopregnant mesenteric resistant arteries. Mean ± S.E.M. of three to four rats. Responses to Yoda1 were analyzed using a two-way ANOVA test. * *p* < 0.05 compared to respective controls.

**FIGURE 4:**
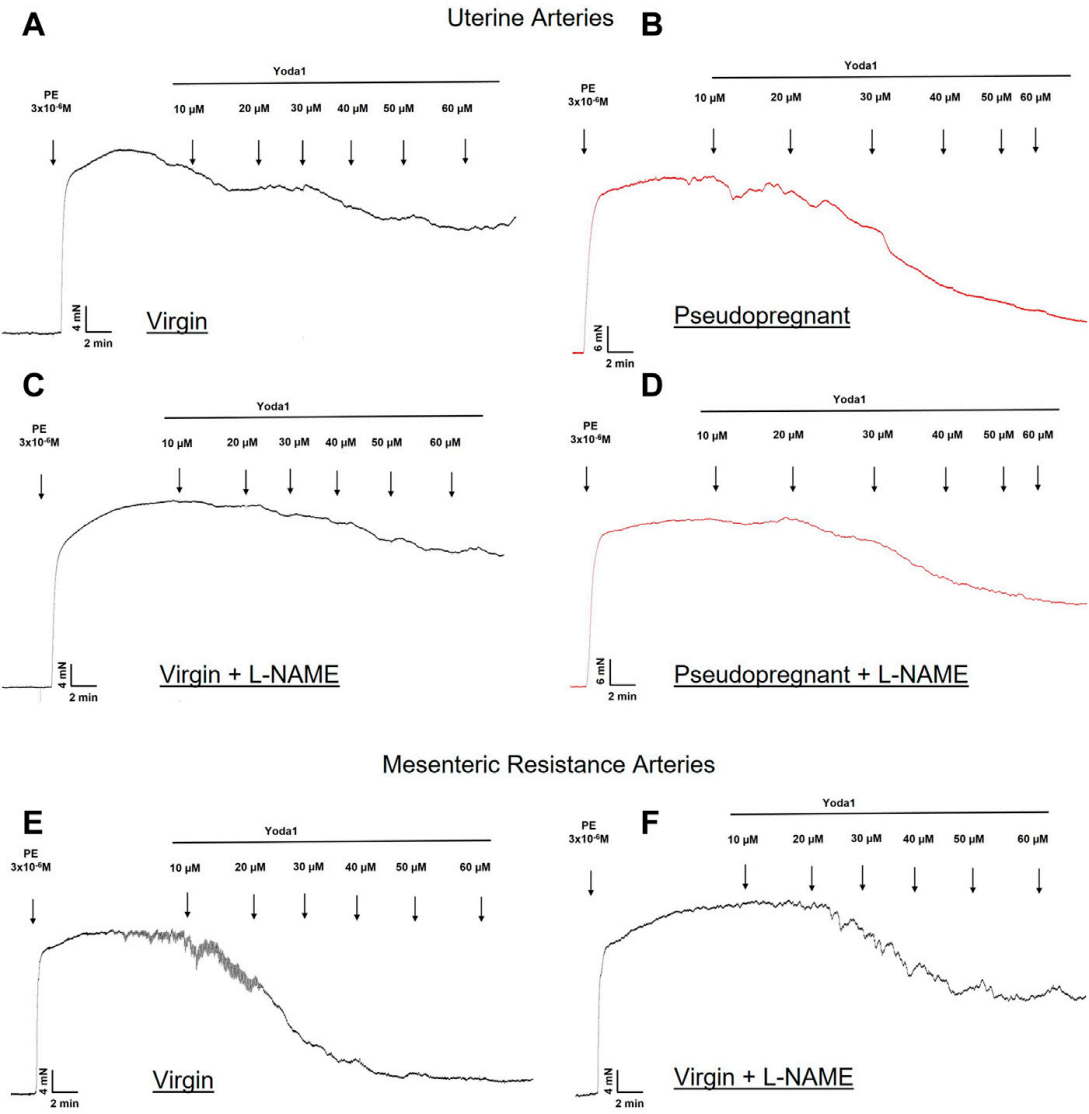
Representative tracing of the relaxation induced by Yoda1 in the UA **(A–D)** and MRA **(E–F)** of virgin **(A–F)** and pseudopregnant rats **(B–D)**. Figures C, D and F represent the relaxation of Yoda1 in the presence of L-NAME (10^−4^ M).

We hypothesize that the increased vasorelaxation response to Yoda1 is nitric oxide dependent. To test this, we performed concentration-response curves to Yoda1 in the presence of the nitric oxide synthase inhibitor (L-NAME 10^−4^ M, 30 min). Our results (Figure 3) show that L-NAME impaired the concentration-dependent relaxation responses to Yoda1, in the UA (Figure 3A, C) and MRA (Figure 3B, D) of the rats, confirming that this response is at least in part mediated by nitric oxide. Figure 4 is the representative tracing of Yoda1-induced relaxation in the uterine arteries of virgin (black) or pseudopregnant (red), in the absence (top panels) or presence (center panels) of L-NAME. The representative tracing of Yoda1-induced relaxation in MRA of virgin rats, in the absence (left panel) or in the presence (right panel) are presented in the bottom panels of Figure 4.

#### 3.1.3 The Yoda1 induced vasorelaxation is not influenced by potassium channel inhibitors

**FIGURE 5:**
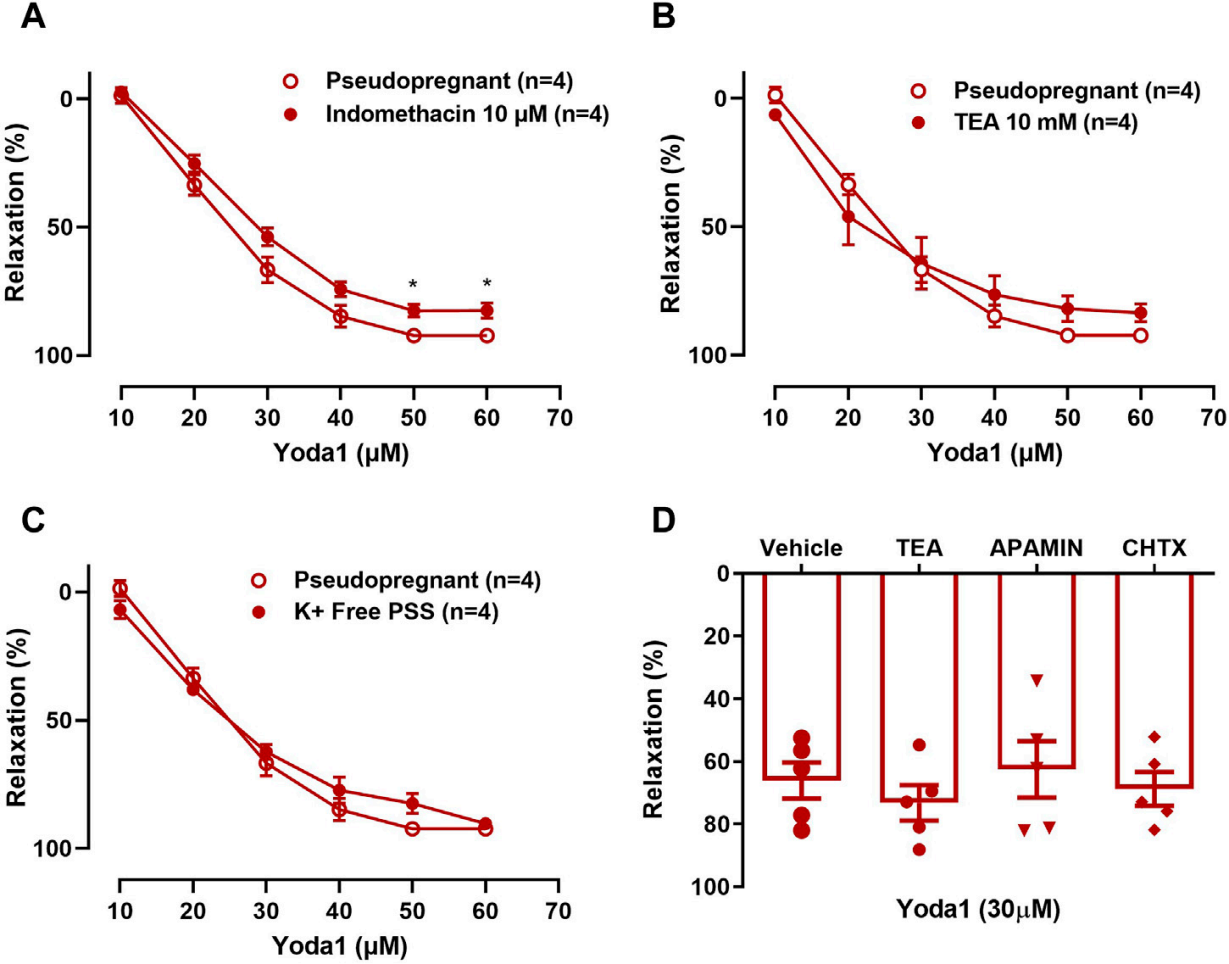
A–C: Relaxation responses to Yoda1 [10^−5^ M—6 × 10^-5^ M] in UA before and after pre-incubation with **(A)** 10^−5^ M indomethacin; **(B)** 2 × 10^-3^ M TEA; **(C)** K^+^-free PSS and **(D)** Relaxation responses to a single concentration of Yoda1 [3 × 10^−5^ M] in UA incubated with 10^−10^ M ChTX, 10^−9^ M Apamin, and 2 × 10^-3^ M TEA. Vessels were pre-contracted with 3 × 10^−6^ M phenylephrine. Mean ± S.E.M. of four to five rats. Responses to Yoda1 in the presence or absence of indomethacin, TEA, or K + -free PSS were analyzed using Student’s t-test. *p < 0.05 compared to the respective control.

In UAs, the relaxation induced by Yoda1 [10^−5^ M—6 × 10^−5^ M] was not affected by indomethacin (COX inhibitor; Figure 5A), TEA (non-selective potassium channel inhibitor; Figure 5B, D), ChTX (IK_Ca_ blocker; Figure 5D), or apamin (SK_Ca_ blocker; Figure 5D).

No changes were seen when the concentration-response curve to Yoda1 was obtained in K^+^-free PSS (Figure 5C), excluding the involvement of the sodium-potassium pump (Na⁺/K⁺-ATPase) in the Yoda1-induced vasorelaxation of the UA.

#### 3.1.4 Concentration-dependent relaxation responses to Yoda1 were greater in UAs of untreated rats compared to those treated with ODN2395

**FIGURE 6:**
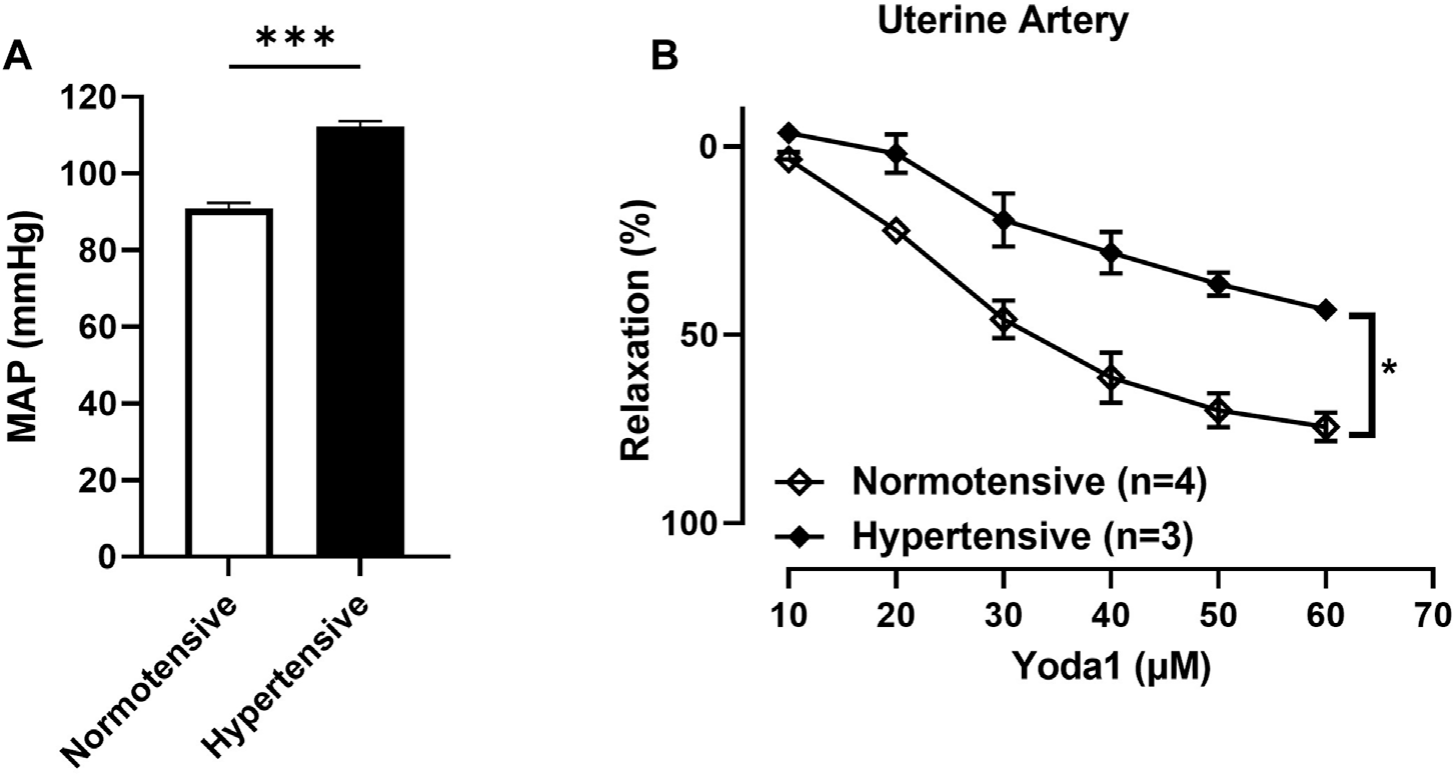
**(A)** Evaluation of the MAP of pregnant rats treated with ODN2395 vs. untreated pregnant rats (112 ± 1 vs. 90 ± 1 *p* = 0.0004). **(B)** Evaluation of concentration-dependent relaxation responses of uterine arterial ring segments to Yoda 1 [10^−5^ M—6 × 10^−5^ M]. Vessels were pre-contracted with 3 × 10^-6^ M Phenylephrine. The Student’s t-test was used to compare the 2 groups. ***p < 0.01; *p < 0.05 compared to pregnant normotensive controls.

Our results showed that the concentration dependent relaxation responses to Yoda1 are greater in UAs of untreated rats compared to those treated with ODN2395 (Figure 6B).

##### Mean arterial pressure was greater in rats treated with ODN2395 vs. untreated rats

We observed a significant difference in the MAP (mmHg) of the pregnant rats treated with ODN2395 compared to the untreated pregnant rats (112.1 ± 1.487 vs. 90.98 ± 1.411 *p* = 0.0004; Figure 6A).

## 4 Discussion

During pregnancy, there is an increase in blood flow in the uterine arteries which leads to increased shear stress (Julian et al., 2009). The endothelial cells lining the arterial wall sense this increase in shear stress and a series of downstream mechanisms are initiated to cause vasodilation. Subsequently, the vasculature undergoes functional and structural remodeling in order to accommodate the increase in blood flow (Koga et al., 2003). This remodeling is not just important for the maintenance of adequate blood flow during pregnancy, if this remodeling does not take place or if there is aberrant remodeling, it leads to a condition known as preeclampsia. Preeclampsia is a condition that usually occurs in the second half of pregnancy-starting at about 20 weeks, during labor, or early *postpartum*. It is characterized by hypertension, proteinuria, organ dysfunction in the pregnant woman, and a decline in fetal growth. It affects 3%–10% of all pregnancies and contributes to fetal and maternal morbidity and mortality in pregnancy (Roberts et al., 1989; Perry et al., 2018). It correlates with increased risk of cardiovascular disease later in maternal life. Preeclampsia is a multisystem disease, and its underlying cause is still unknown, though it is known to begin with aberrant placentation, and vascular remodeling. However, the exact mechanism responsible for the aberrant placentation and how it leads to cardiovascular disease remains currently unclear.

The main source of uterine vascular resistance is the highly muscular spiral arteries. They are the resistance vessels that contract and relax to modulate blood flow in response to changing uterine metabolic needs. During pregnancy, the syncytiotrophoblast invades the endometrium and the cytotrophoblast cells become extra villous trophoblast (EVT), which are migratory and non-proliferative cells that invade connective tissues and maternal walls. These EVTs migrate into the spiral arteries and transform them from muscular resistance vessels to wide, flaccid conduits and blood pours into the intervillous space at high pressure (Huppertz et al., 2009). Eradication of the maternal resistance vessels maximizes flow to the placental site but simultaneously limits the vascular control system’s ability to regulate blood flow. This flow becomes a direct function of uterine arterial pressure as predicted by the hemodynamic equivalent of Ohm’s law (Venkatesha et al., 2006). The uterine vascular remodeling that occurs in normal pregnancy is absent in preeclampsia. Cytotrophoblast invasion of the spiral arteries is poor leading to decreased uteroplacental perfusion and consequently preeclampsia. The spiral arteries remain a small resistance vessel. A study of basal plates of placentas of abnormal pregnancies confirmed that the remodeling of the spiral arteries that occurs in normal pregnancy was completely absent in preeclampsia (Khong et al., 1986). Although the initiating event is thought to be decreased uteroplacental perfusion as a result of aberrant vascular remodeling, the main cause of this aberrant remodeling is currently unknown. There is therefore a need to understand the mechanism by which changes in flow of the uterine artery in pregnancy activates endothelium-dependent vasodilation. This will pave the way for new insights into understanding the mechanisms of vascular adaptations that occur in the uterine arterial bed, improving pregnancy outcomes and ultimately, later health life.

First, we tested the hypothesis that the shear-stress-associated mechanosensitive Piezo1 channel is upregulated in the uterine arteries during pregnancy, we did this by evaluating the concentration-dependent relaxation responses of uterine arterial ring segments to the chemical activator of the Piezo1 channel, Yoda1. Our results show that concentration-dependent relaxation responses to Yoda1 are greater in uterine arterial ring segments of pseudo-pregnant rats compared to virgin rats. Yoda1 chemically activates the Piezo1 channel and mimics the effects of shear stress on endothelial cells (Syeda et al., 2015). The Piezo1 channel is important for mechanotransduction in response to shear stress in the endothelial cell (Ranade et al., 2014). Mechanotransduction, which is the process through which mechanical force is converted to a physiological signal is very important in the maintenance of blood flow and tone (Humphrey et al., 2015). During pregnancy, uterine arterial blood flow increases significantly. Despite this increase in blood flow, blood pressure remains unchanged. This is accomplished by a decrease in the vascular resistance of the uterine artery brought about by vasodilation of the artery (Osol and Mandala, 2009). Other mechanisms have been proposed for the increase in blood flow during pregnancy, but the maintenance of vasodilation in the uterine vasculature is very important and needs to be fully explored. During pregnancy, the hemodynamic adaptation results in decreased uterine artery resistance and increased blood flow. This increase in blood flow will increase flow velocity increasing shear stress. An increase in shear stress then stimulates vasodilation through a mechanism known as ‘shear-stress-induced vasodilation. Studies have suggested that this shear-stress-induced vasodilation is endothelial in origin and that NO, which is a potent vasodilator plays a key role in this process (Bird et al., 2003). Several studies have provided evidence to support the role of NO in uterine arterial adaptation during pregnancy including a study that showed that UA remodeling was attenuated in pregnant eNOS-deficient mice and pregnant rats who were given a NOS inhibitor (Osol et al., 2009). Furthermore, increases in the synthesis and levels of NO have been reported. Earlier studies had demonstrated that there is an increase in the endogenous biosynthesis of NO during pregnancy and pseudopregnancy (Conrad et al., 1993). Another study reported that uterine arterial eNOS protein and mRNA expression increase during pregnancy (Magness et al., 2001). The question is what is responsible for this increase in NO-mediated vasodilation? We used pseudopregnant rats in our study because pseudopregnant rodents have been used as a pregnancy-like model, which can be manipulated without terminating the pregnancy or killing the fetuses. Pseudopregnant rats have the same hormonal profile and vascular hemodynamic adaptations as pregnant rats. (Bui et al., 1986; van der Heijden et al., 2005; Augustine and Grattan, 2008; Augustine et al., 2019).

Our results show that concentration-dependent relaxation responses to Yoda1 are greater in uterine arterial ring segments of pseudopregnant rats compared to virgin rats. There are no differences observed in the concentration-dependent relaxation responses to Yoda1 in mesenteric resistance arterial ring segments of pseudo-pregnant rats compared to the virgin rats. To confirm if the difference in the relaxation response to Yoda1 was a result of the activation of the Piezo1 channel, we tested the relaxation response of the same vessels to ACh and SNP and we observed no difference in the relaxation responses to ACh and SNP in the UAs of these rats. These results suggest that Piezo1 channels are involved in the dilation that occurs in the UAs of pseudo-pregnant rats. Literature has provided evidence to corroborate this finding. Ranade and his group (Ranade et al., 2014) showed that Piezo1 is a critical component of endothelial cell mechanotransduction and is required for embryonic development. Piezo1 is expressed in embryonic endothelial cells and is activated by fluid shear stress and loss of Piezo1 affects the ability of endothelial cells to alter their alignment when subjected to shear stress (Rode et al., 2017). Another study showed that Piezo 1 is required for flow-induced ATP release and subsequent P2Y_2_/G_q_/G_11_–mediated activation of downstream signaling that results in phosphorylation and activation of Protein Kinase B (AKT) and endothelial NOS (Wang et al., 2016). They also found that mice with induced endothelium-specific Piezo 1 deficiency lost the ability to induce NO formation and vasodilation in response to flow and consequently developed hypertension (Wang et al., 2016). Endogenous Piezo 1 channels in the endothelium have been suggested to be direct sensors of shear stress by a study which showed activation by shear stress in excised outside-out cell-free membrane patches. Piezo1 channels have also been shown to be expressed in vascular smooth muscle cells of small-diameter arteries and play a role in the structural remodeling (John et al., 2018). Furthermore, a previous study from our lab showed that Piezo1 channels are expressed in the pudendal arteries and corpus cavernosum and that its activation by Yoda1 leads to relaxation of the pudendal artery and the corpus cavernosum (Dela Justina et al., 2023).

Next, we wanted to know if the concentration-dependent relaxation responses to Yoda1 in uterine arterial ring segments of pseudopregnant rats are nitric oxide-dependent. Our results show that L-NAME impaired the relaxation responses to Yoda1, in the UA and MRA of the rats. Activation of the Piezo1 channel results in an increase in intracellular Ca^2+^ levels (Syeda et al., 2015). Also, chemical stimulation of the Piezo1 channel by the exogenous agonist, Yoda1 elicits Ca^2+^ flux in Piezo1 transfected cells and it is dependent on extracellular Ca^2+^ (Syeda et al., 2015). Furthermore, concentration-response experiments showed that Yoda1 at micromolar concentrations induced robust Ca^2+^ responses in cells transfected with either human or mouse Piezo1 (Cox et al., 2016). We can therefore infer that in the uterine arterial endothelial cells of the pseudopregnant rats, activation of Piezo1 channel stimulates Ca^2+^ influx, increasing endothelial Ca^2+^ leading to activation of eNOS and subsequent release of NO, which causes vasorelaxation.

The inhibition of NO production with L-NAME did not completely abolish the relaxation response to Yoda1. Therefore, we tested the hypothesis that other mechanisms are underlying the vasorelaxation response to the activation of the Piezo1 channel by Yoda1. First, we investigated the role of cyclooxygenase (COX) in the relaxation response of the uterine arteries to Yoda1. COX which is one of the main enzymes in the production of prostaglandins is expressed in the endothelium of the UAs and this expression is greater in the endothelium than in the vascular smooth muscle cells. Furthermore, COX expression is increased during pregnancy (Janowiak et al., 1998). We did this by incubating the arterial rings with indomethacin. Indomethacin is a potent, non-selective inhibitor of both COX-1 and COX-2 (Blobaum et al., 2013). Our result showed that indomethacin impaired the relaxation response to Yoda1. This suggests that COX and the prostaglandin pathway play a role in the Piezo1 mediated vasodilation in the UAs of rats.

Next, since the Piezo1 channel is an ion channel we investigated whether K^+^ channels have a role in the relaxation response to Yoda1 in the UA of pseudopregnant rats. Using TEA, we blocked the K^+^ channels. Our results showed no differences in the relaxation responses to Yoda1 in the presence or absence of TEA. We also tested the effects of small and intermediate conductance Ca^2+^ activated K^+^ channel (SK_Ca_ and IK_Ca_) in the relaxation response to Yoda1. The SK_Ca_ and IK_Ca_ are expressed in the endothelial cells and these channels contribute to endothelial NO generation and lead to vasorelaxation (Félétou, 2009). Activation of these channels also leads to endothelium-dependent hyperpolarization of small resistance arteries (Gluais et al., 2005) contributing to the agonist-mediated vasorelaxation responses. To do this, we incubated the UA with blockers of IK_Ca_, ChTX, and of SK_Ca,_ Ap for 30 min and stimulated the vessels (pre-contracted with PE) with a single dose of Yoda1. Our results showed that these channels are not involved in the Piezo1 mediated vasodilation of UA from pseudopregnant rats. Other ion channels could be involved including stretch-activated ion channels, which have been described in endothelial cells and it has been suggested that they could be involved in the response to mechanical forces generated by blood flow and pressure (Lansman et al., 1987). Other studies proposed that stretch-activated channels are responsible for detection of mechanical stimulus (Naruse et al., 1998) and the reorientation response to mechanical stress seen in endothelial cells was prevented by chemical inhibitors of these ion channels (Naruse et al., 1998). However, the identity and mechanism through which they elicit their response remains unknown. Another study suggested that a K^+^ selective ionic current is involved in the process of mechanotransduction (Olesen et al., 1988). Additionally, it has been established that shear stress-induced Ca^2+^ influx activates downstream signaling and this has led to the suggestion that ion channels are responsible for the mechanotransduction of shear stress in the endothelial cells.

Piezo1 channels are also permeable to Na^+^ (Coste et al., 2010). Therefore we explored the role of Na^+^ in the Piezo1-mediated vasodilation of UA from pseudopregnant rats. First, we tested the role of Na⁺/K⁺-ATPase in the Yoda1-induced relaxation by performing the concentration-response curve to Yoda1 in the presence of K^+^-free PSS. In the vasculature, Na⁺/K⁺-ATPase contributes to the regulation of transmembrane potential and vascular tone (Blaustein, 1988). Na⁺/K⁺-ATPase inhibition using K^+^-free PSS leads to membrane depolarization and Ca^2+^ influx *via* voltage-sensitive Ca^2+^ channels (Ofoh et al., 2014). Our results show no differences in the concentration-dependent relaxation responses to Yoda1, in the absence or presence of K^+^-free PSS in the uterine arterial ring segments of the rats.

The first contribution of this study to our knowledge is that concentration-dependent relaxation responses to Yoda1 are greater in uterine arterial ring segments of pseudo-pregnant rats compared to virgin rats. Secondly, our results show that concentration-dependent relaxation responses to Yoda1 are partially mediated by the endothelial NO, which is already known as John et al., reported this in 2018 (John et al., 2018). However, inhibition of NO production did not completely abolish the relaxation response to Yoda1 in their study and ours also. Therefore, we tested the hypothesis that other mechanisms are underlying the vasorelaxation response to the activation of the Piezo1 channel by Yoda1. Our findings show that the K^+^ channels are not involved in the Piezo1-mediated vasorelaxation of UA from pseudopregnant rats. We also showed that the Na⁺/K⁺-ATPase is not involved in the Piezo1 channel-mediated relaxation of the UAs from rats. Finally, we report that relaxation responses to Yoda1 are impaired in the uterine arteries of hypertensive pregnant rats, thereby suggesting that the Piezo1 channel might be involved in the vasodilation of uterine arteries of pregnant rats.

## 5 Conclusion

These results demonstrate that the Piezo1 channel is involved in the dilation that occurs in the UAs of pseudo-pregnant rats through, at least in part, a NO-dependent mechanism, while in pregnant rats, Piezo1 activation is impaired in hypertension. More studies need to be done to understand whether changes in flow during pregnancy play a role in the activation of Piezo1 in the uterine arteries. This will pave the way for new insights into understanding the mechanisms of vascular adaptations that occur in the uterine arterial bed, improving pregnancy outcomes. Moreover, given the character of this channel and its contribution to the relaxation of UA in pregnancy, further studies are needed to elucidate whether a dysfunctional Piezo1 might be implicated in the pathophysiology of preeclampsia.

## 6 Study limitation

The major limitation of our study is the unavailability of appropriate tools and techniques to study the mechanism of action of the Piezo1 channel, particularly the fact that there is no selective inhibitor of the channel. We also could not obtain calcium measurements in the bath solution.

## Data Availability

The original contributions presented in the study are included in the article/supplementary material, further inquiries can be directed to the corresponding author.
